# Enhancement of immune maturation in suckling rats by leptin and adiponectin supplementation

**DOI:** 10.1038/s41598-018-38418-1

**Published:** 2019-02-11

**Authors:** Blanca Grases-Pintó, Mar Abril-Gil, Margarida Castell, Francisco J. Pérez-Cano, Àngels Franch

**Affiliations:** 1 Physiology Section, Department of Biochemistry and Physiology, Faculty of Pharmacy and Food Science, University of Barcelona, 08028 Barcelona, Spain; 2 Nutrition and Food Safety Research Institute (INSA·UB), 08921 Santa Coloma de Gramenet, Spain

## Abstract

Leptin and adiponectin, adipokines present in breast milk, have shown immunomodulatory properties. The current study aimed to ascertain whether a nutritional supplementation with leptin or adiponectin in neonatal rats was able to influence the maturation of the systemic immune response in early life. To achieve this, suckling Wistar rats were supplemented with either leptin (0.7 μg/kg/day) or adiponectin (35 μg/kg/day) during the whole suckling period. Plasmatic immunoglobulins were quantified, and spleen lymphocyte composition and their ability to proliferate and release cytokines were evaluated during (day 14) and at the end (day 21) of the suckling period. Rats fed with either adipokine showed higher plasma IgM and IgG1 concentrations and adiponectin supplementation also increased IgG2a at both studied days (P < 0.05). With regard to the lymphocyte composition, both adipokine supplementations increased T cell proportion and both CD4^+^ and CD8^+^ T cell subsets after two weeks of supplementation (P < 0.05). Moreover, only leptin administration increased NK and NKT cell proportions at the end of the suckling period. Finally, both adipokines influenced the cytokine secretion pattern by splenocytes. In conclusion, these results suggest that leptin and adiponectin play a role in the maturation of the systemic immune response during the suckling period.

## Introduction

It is well known that the neonatal period is critical because the newborn has to be capable of adapting him/herself to the new environment, such as the source of nutrients from the umbilical cord to breast milk^1^. Moreover, at birth, the baby is exposed to a large number of microorganisms and foreign proteins, and the neonatal immune system (IS) is not yet well prepared to fight them because its innate and acquired immunity is far from that of an adult^2^. The rapid and appropriate development of the IS can prevent a number of neonatal infections. To compensate for the immunological immaturity of the baby, during the gestation the mother gives protection, transferring transplacental antibodies and giving anti-infective resistance factors in the amniotic fluid. In the extrauterine life, breastfeeding provides high concentrations of protective and immunomodulatory factors^3^.

Neonatal nutrition is a critical factor for the development of intestinal and systemic IS^4^. Evidence has shown that breast milk is the ideal food for the newborn during the first months of life, as it is nutritionally adapted to the requirements of the baby. In addition to macro- and micronutrients, human milk contains numerous bioactive factors that promote the development and functionality of the IS as well as other components that protect against infection^5–7^. Thus, breast milk contains antimicrobial components, such as immunoglobulin (Ig) A, lactoferrin, lysozyme, oligosaccharides, gangliosides, etc. Moreover, milk has bioactive factors that contribute to the development and function of the neonatal IS, such as nucleotides, hormones, growth factors and cytokines, among others^5,8^. It also contains maternal leukocytes in an activated phenotype, such as macrophages, neutrophils and lymphocytes^9^. In general, the components found in milk come from the maternal circulation or are synthesized directly in the mammary glands^8^. All these components are hypothesized to play a key role in the pathway between the naïve IS of the infant and the IS of the child^10^ and that is why breastfed children tend to be healthier than formula-fed children^11^.

Leptin and adiponectin are adipokines synthesized mainly by white adipose tissue. Moreover, they are abundant in breast milk and are able to cross the infant’s intestinal wall and to reach his bloodstream^12,13^. It is well known that these adipokines are involved in the control of energy balance, but it is well described that they also possess immune activity^14–16^. The vast majority of the studies on the effect of leptin and adiponectin on the IS are performed *in vitro*^17,18^, and very few have addressed their *ex vivo* or *in vivo* functionality^19^. In addition, there are no studies on the effect of these adipokines on neonatal immune response, and therefore, their impact on the maturation of the IS in early life is not known.

We have recently described the potential of both adipokines to modulate immune development in early life at the intestinal level, where leptin and adiponectin firstly contact with the host defensive system^20^. We hypothesize that these adipokines could also have a role in the development of the IS in early life at the systemic level. The particular aim of the present study is to evaluate whether leptin and adiponectin have a modulatory effect on the systemic humoral immune response and on the spleen cell immune functionality, a secondary lymphoid tissue representative of systemic IS. To this end, newborn rats were supplemented with leptin or adiponectin during the entirety of the suckling period. The immune status was established in plasma by the evaluation of Ig concentration and in the spleen by its lymphocyte phenotypical characterization and its functional ability to proliferate and to secrete cytokines during (day 14) and at the end of suckling period (day 21).

## Results

### Body and lymphoid organs’ weight

Animals supplemented with adipokines showed similar body weight when compared with pups receiving vehicle or Whey Protein Concentrate (WPC) (Table 1). Although there were no differences due to the dietary supplementation on the relative spleen weight, an increase in relative thymus weight was observed in the Adiponectin and WPC groups at the end of the suckling period (P < 0.05 *vs*. Reference group on day 21, Table 1).

**Table 1:** Body weight, relative spleen weight and relative thymus weight from the four groups: Reference, Leptin, Adiponectin and Whey Protein Concentrate (WPC) over the suckling period (day 14 and 21).

		Body weight (g)	Relative spleen weight (%)	Relative thymus weight (%)
Day 14	Reference	37.45 ± 0.60	0.61 ± 0.02	0.38 ± 0.02
	Leptin	38.04 ± 1.97	0.55 ± 0.03	0.39 ± 0.02
	Adiponectin	34.32 ± 0.75	0.61 ± 0.03	0.44 ± 0.04
	WPC	36.06 ± 1.69	0.65 ± 0.03	0.39 ± 0.02
Day 21	Reference	63.19 ± 1.42^Ψ^	0.61 ± 0.03	0.39 ± 0.02
	Leptin	62.56 ± 3.02^Ψ^	0.62 ± 0.02^Ψ^	0.43 ± 0.02
	Adiponectin	61.18 ± 1.75^Ψ^	0.62 ± 0.03	0.46 ± 0.01*
	WPC	59.62 ± 1.86^Ψ^	0.68 ± 0.03	0.46 ± 0.02*^Ψ^

The results are expressed as a mean ± S.E.M. (n = 9−12). Statistical differences: *P < 0.05 *vs*. Reference group. ^Ψ^P < 0.05 *vs*. same group data at day 14.

### Plasma IgA, IgM and IgG concentrations

**Figure 1:**
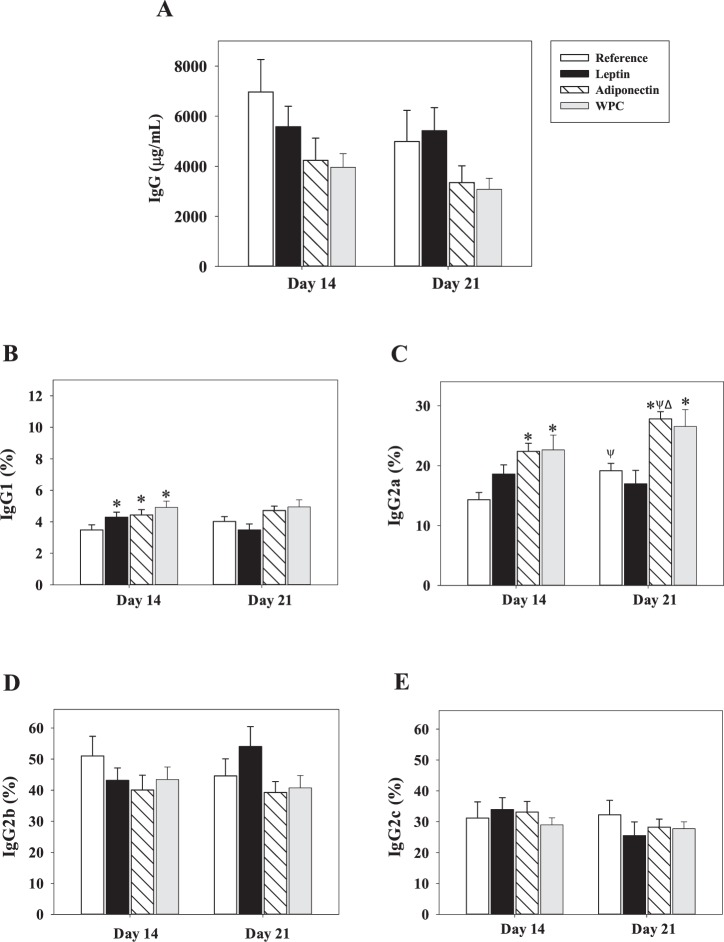
Plasma IgG concentration after adipokine supplementation. Effect of dietary supplementation with adipokines on the concentration of IgG and their subclasses in plasma from the four groups − Reference, Leptin, Adiponectin and Whey Protein Concentrate (WPC) − over the suckling period (day 14 and 21). IgG (**A**), IgG1 (**B**), IgG2a (**C**), IgG2b (**D**) and IgG2c (**E**). The IgG subclasses were calculated as the percentage of each particular isotype concentration with respect to the total IgG concentration. The results are expressed as a mean ± S.E.M. (n = 9−12). Statistical differences: ^*^P < 0.05 *vs*. Reference group. ^Δ^P < 0.05 *vs*. Leptin group. ^Ψ^P < 0.05 *vs*. same group data at day 14.

**Figure 2:**
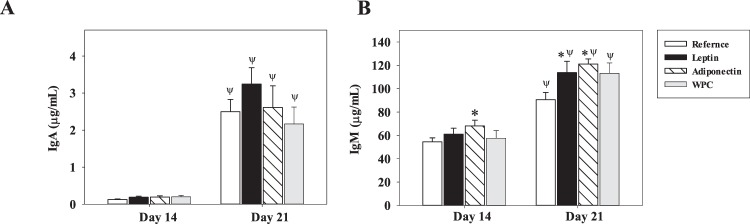
Plasma IgA and IgM concentrations after adipokine supplementation. Effect of dietary supplementation with adipokines on plasmatic IgA (**A**) and IgM (**B**) concentrations from the four groups − Reference, Leptin, Adiponectin and Whey Protein Concentrate (WPC) − over the suckling period (day 14 and 21). The results are expressed as a mean ± S.E.M. (n = 8−12). Statistical differences: *P < 0.05 *vs*. Reference group. ^Ψ^P < 0.05 *vs*. same group data at day 14.

To assess the influence of the adipokine supplementations on the production of antibodies in the systemic compartment, IgA, IgM, IgG and IgG isotypes (IgG1, IgG2a, IgG2b, IgG2c) were quantified in plasma samples during (day 14) and at the end of the suckling period (day 21) (Figs. 1 and 2).

IgG is the most abundant Ig in plasma (Fig. 1), followed by IgM and finally IgA (Fig. 2), and with regard to IgG isotypes, the Th1-related IgG (IgG2b and IgG2c) were present in higher proportions than the Th2-related ones (IgG1 and IgG2a) (Fig. 1). Age-associated increases (between 14- and 21-d-old rats) were observed in all groups for IgA and IgM (P < 0.001, Fig. 2) but not for IgG (Fig. 1). However, within IgG, the 14 to 21 days age-associated changes were only observed in IgG2a levels in the Reference and Adiponectin groups (P < 0.01, Fig. 1C).

With reference to IgG, the three dietary supplementations did not modify their concentration in any of the two studied days (Fig. 1A). However, adiponectin and WPC supplementations significantly increased the proportion of Th2-related Ig isotypes without affecting that of the Th1-related ones. Specifically, the pups plasma of Adiponectin and WPC groups had a higher percentage of IgG1 than the Reference group at day 14 (P < 0.05) and IgG2a at both days of the study (P < 0.01, Figs. 1B,C). Leptin supplementation was only able to increase IgG1 abundance at day 14 (P < 0.05, Fig. 2B).

With regard to plasma IgA and IgM concentrations (Fig. 2), whereas no changes were found in IgA levels due to the diets, after 3 weeks of leptin or adiponectin supplementation, an increase in plasma IgM concentration was observed compared with the Reference group (P < 0.05 and P < 0.01, respectively). In the case of adiponectin, this significant increase could already be seen at day 14 (P < 0.05, Fig. 2B).

### Lymphocyte composition of spleen

**Figure 3:**
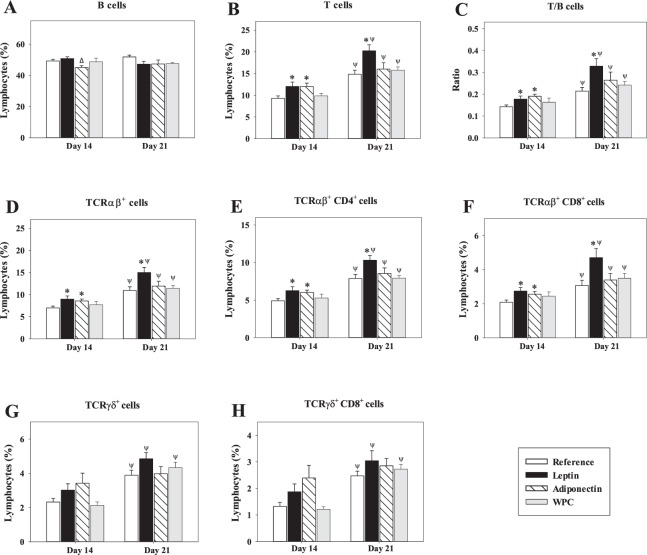
Main spleen cell subsets after adipokine supplementation. Effect of dietary supplementation with adipokines on the main splenocyte subsets from the four groups − Reference, Leptin, Adiponectin and Whey Protein Concentrate (WPC) − over the suckling period (day 14 and 21). B cells defined as CD45RA^+^ CD4^−^ CD8^−^ cells (**A**), T cells defined as the sum of TCRαβ^+^ NKR-P1A^−^ and TCRγδ^+^ cells (**B**), ratio T/B cells (**C**), TCRαβ^+^ NKR-P1A^−^ cells (**D**), TCRαβ^+^ CD4^+^ CD8^−^ cells (**E**), TCRαβ^+^ CD8^+^ CD4^−^ cells (**F**), TCRγδ^+^ cells (**G**) and TCRγδ^+^ CD8^+^ CD4^−^ cells (**H**). The results are expressed as a mean ± S.E.M. (n = 8−12). Statistical differences: *P < 0.05 *vs*. Reference group. ^Δ^P < 0.05 *vs*. Leptin group. ^Ψ^P < 0.05 *vs*. same group data at day 14.

The percentages of B cells, T cells and their subsets TCRαβ^+^, TCRαβ^+^ CD4^+^, TCRαβ^+^ CD8^+^, TCRγδ^+^ and TCRγδ^+^ CD8^+^ in the lymphocyte population selected according to their forward-scatter characteristics (FSC) and side-scatter characteristics (SSC) from all experimental groups throughout the study are summarised in Figure 3.

Although there was not any significant change in B cell population between the three supplemented groups and the Reference group, a punctual decrease on day 14 was observed in rats supplemented with adiponectin compared with the Leptin group (P < 0.01, Fig. 3A). Both adipokine supplementations caused higher T-cell percentage than the Reference group at day 14 (P < 0.05 in the Leptin group and P < 0.01 in the Adiponectin group, Fig. 3B). This effect was also observed in the case of the Leptin group at day 21 (P < 0.01 *vs*. the Reference group, Fig. 3B). These increases in T cells induced a significant increase in the T/B cells ratio (P < 0.05, Fig. 3C). Conducting a deeper study into which subpopulation of T cells was responsible for this increase, on both studied days we found that there was a higher percentage of TCRαβ^+^ cells in the adipokine-supplemented animals compared with that of the animals from the Reference group (P < 0.01, Fig. 3D), and more specifically an increase in both TCRαβ^+^ CD4^+^ and TCRαβ^+^ CD8^+^ cell percentages (P < 0.01 *vs*. Reference group, Figs. 3E,F). On the other hand, all groups showed an age-increasing proportion of T, TCRαβ^+^, TCRαβ^+^ CD4^+^ and TCRαβ^+^ CD8^+^ cells and also the T/B cells ratio (P < 0.01, Figs. 3B−F).

Despite no significant changes in TCRγδ^+^ and TCRγδ^+^ CD8^+^ proportions due to the supplementations, the Reference, Leptin and WPC groups, but not the Adiponectin group, significantly increased the proportion of these populations compared with percentages of these groups on day 14 (P < 0.05), because in the Adiponectin group similar proportion of that on day 21 had already been achieved on day 14 (Figs. 3G,H).

Moreover, Treg subset (CD4^+^ CD25^+^ Foxp3^+^) was also studied and neither the age nor the dietary supplementations significantly modified their percentages, ranging in all cases from 0.38% to 0.73%.

**Figure 4:**
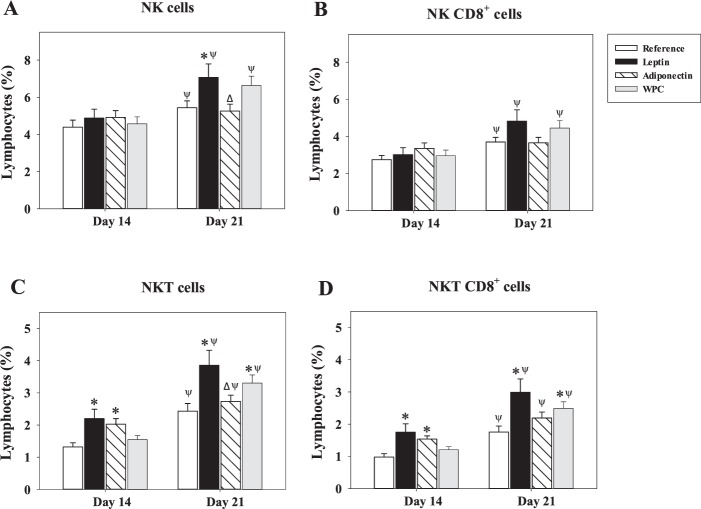
NK and NKT cell subsets after adipokine supplementation. Effect of dietary supplementation with adipokines on the NK defined as NKR-P1A^+^ TCRαβ^−^ (**A**) and NKT defined as NKR-P1A^+^ TCRαβ^+^ (**C**) splenocyte subsets and their expression of CD8 co-receptor (NK CD8^+^ (**B**) and NKT CD8^+^ (**D**)) from the four groups − Reference, Leptin, Adiponectin and Whey Protein Concentrate (WPC) − over the suckling period (day 14 and 21). The results are expressed as a mean ± S.E.M. (n = 8−12). Statistical differences: *P < 0.05 *vs*. Reference group. ^Δ^P < 0.05 *vs*. Leptin group. ^Ψ^P < 0.05 *vs*. same group data at day 14.

Furthermore, the study of NK, NKT cell percentage and their expression of CD8 co-receptor was also performed (Fig. 4). Overall, and with the only exception being the NK subset in animals supplemented with adiponectin, the proportion of NK and NKT cells increased with age in all groups (P < 0.05 day 21 *vs*. day 14, Figs. 4A,C). In agreement with this, the proportion of these cells co-expressing CD8, which was 65−75% for both subsets, led to an age-dependent increase, being the percentage of NK and NKT subpopulations expressing CD8 co-receptor higher at day 21 than at day 14 (P < 0.05), again with the exception of the Adiponectin group in NK cells (Figs. 4B,D). After 3 weeks of nutritional intervention, the rats supplemented with leptin showed an increase in the proportion of NK cell population (P < 0.05 *vs*. Reference and Adiponectin groups, Fig. 4A), which could not be significantly associated with changes in CD8 co-expression (Fig. 4B). Both adipokine supplementations were able to increase NKT percentage at day 14 (P < 0.05 and P < 0.01 *vs*. Reference group, respectively), to a proportion similar to that found in Reference animals on day 21 (>2%, Fig. 4C). At day 21, this change was still detected in the Leptin group with respect to the Reference and Adiponectin groups (P < 0.01 and P < 0.05, respectively) and, it was also significantly higher in the WPC group (P < 0.01 *vs*. Reference group, Fig. 4C).

**Figure 5:**
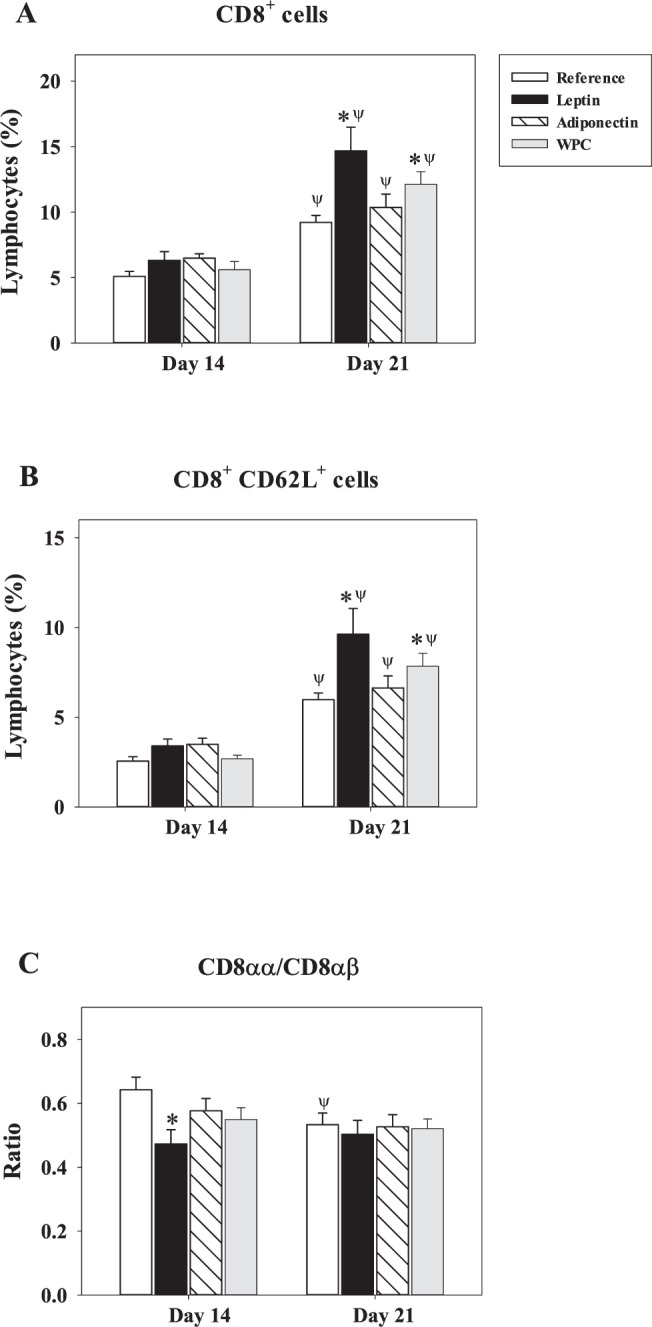
Changes in CD8^+^ cell subsets after adipokine supplementation. Effect of dietary supplementation with adipokines on the CD8^+^ (**A**), CD8^+^ CD62L^+^ (**B**) splenocyte subsets and CD8αα/CD8αβ ratio (**C**) from the four groups − Reference, Leptin, Adiponectin and Whey Protein Concentrate (WPC) − over the suckling period (day 14 and 21). The results are expressed as a mean ± S.E.M. (n = 8−12). Statistical differences: *P < 0.05 *vs*. Reference group. ^Ψ^P < 0.05 *vs*. same group data at day 14.

Overall, spleen CD8^+^ lymphocytes proportion, used as an immune-maturation biomarker, increased with age in all groups, with the percentage at day 21 being approximately twice as high as that on day 14 (P < 0.01, Fig. 5A). In addition, although no effect was found at day 14, leptin and WPC supplementations induced a higher percentage compared with the Reference group at day 21 (P < 0.01, Fig. 5A). This pattern corresponded to the population of CD8^+^ presenting the adhesion molecule CD62L selectin in their membrane (Fig. 5B). Further analysis in CD8^+^ cells showed a punctual increase of 50% of CD8αβ population at day 14 in those rats supplemented with leptin (P < 0.05 *vs*. Reference group), which induced a decrease in the CD8αα/CD8αβ ratio (P < 0.05 *vs*. Reference group, Fig. 5C). Animals from the Reference group were the only ones that presented statistical differences between day 14 and day 21 in this ratio (P < 0.05) because the supplemented groups at day 14 already had similar levels to those of day 21 (Fig. 5C).

The proportion of cells expressing the homing αE integrin (CD103) (ranging from 0.86−1.16%), the activation marker CD25 (ranging from 1.18−1.85%) and the toll-like receptor 4 (TLR-4) (ranging from 0.21−0.81%) were also quantified, but there were no significant differences among the groups either at day 14 or day 21 nor was an age-associated pattern found.

### Splenocyte proliferation and cytokine production

**Figure 6:**
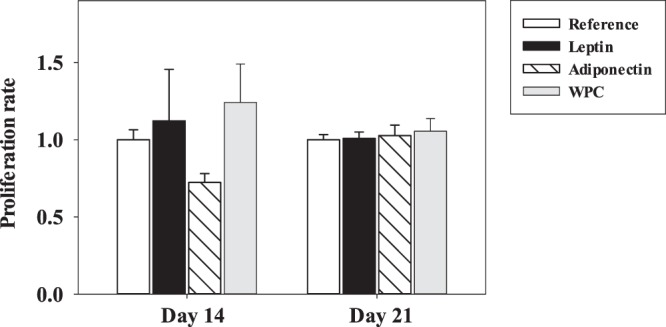
Splenocyte proliferation after adipokine supplementation. Effect of dietary supplementation with adipokines on proliferation rate in mitogen-stimulated splenocytes from the four groups − Reference, Leptin, Adiponectin and Whey Protein Concentrate (WPC) − over the suckling period (day 14 and 21). The results are expressed as a mean ± S.E.M. (n = 3−12 pups per group analysed in quadruplicate).

Splenocytes obtained from suckling rats at days 14 and 21 were stimulated for 48 h and their ability to proliferate (Fig. 6) and secrete cytokines was determined (Table 2). No statistically significant differences were achieved in the lymphoproliferative response at either of the two studied days (Fig. 6). However, some changes were found in the cytokine production due to adipokine supplementation (Table 2).

**Table 2:** Cytokine pattern secreted by splenocytes from the four groups: Reference, Leptin, Adiponectin and Whey Protein Concentrate (WPC) over the suckling period (day 14 and 21).

Cytokines (pg/mL)	Day 14			
	Reference	Leptin	Adiponectin	WPC
IL-2	348.92 ± 63.45	352.52 ± 104.37	600.95 ± 183.86	343.96 ± 42.48
IL-4	69.90 ± 9.64	202.85 ± 61.59*	91.82 ± 14.21	71.60 ± 13.36^Δ^
IL-10	148.92 ± 10.67	196.88 ± 53.91	364.38 ± 91.44*	222.87 ± 46.17
IL-12p70	179.15 ± 48.41	110.42 ± 39.27	216.42 ± 28.44	335.67 ± 123.88
IFN-γ	381.65 ± 92.64	1379.88 ± 679.35	1834.42 ± 338.28*	400.22 ± 31.33^Φ^
TNF-α	15.81 ± 3.93	43.46 ± 19.28	29.26 ± 6.87	20.52 ± 1.64
	**Day 21**			
IL-2	185.90 ± 41.54^Ψ^	286.66 ± 16.59*	139.84 ± 43.46^ΔΨ^	222.43 ± 53.99
IL-4	35.09 ± 7.78^Ψ^	71.59 ± 9.95*^Ψ^	35.54 ± 10.24^Ψ^	52.27 ± 12.70
IL-10	188.52 ± 12.75	239.88 ± 10.69*	185.99 ± 18.31^Ψ^	204.16 ± 9.91^Δ^
IL-12p70	283.64 ± 13.04^Ψ^	317.26 ± 25.59^Ψ^	296.60 ± 37.97	353.58 ± 24.76*
IFN-γ	802.84 ± 172.31	1360.82 ± 267.48	801.19 ± 170.70^Ψ^	1117.86 ± 220.70
TNF-α	30.54 ± 6.26	48.52 ± 5.08	28.62 ± 8.58	38.21 ± 8.79

The results are expressed as a mean ± S.E.M. (n = 9–12). The quantitative determinations were performed with the following limits of detection: 2.10 pg/mL for IL-2; 0.85 pg/mL for IL-4; 14 pg/mL for IL-10; 4.93 pg/mL for IL-12p70; 4.35 pg/mL for IFN-γ; and 3.08 pg/mL for TNF-α. Statistical differences: *P < 0.05 *vs*. Reference group. ^Δ^P < 0.05 *vs*. Leptin group. ^Φ^P < 0.05 *vs.* Adiponectin group. ^Ψ^P < 0.05 *vs*. same group data at day 14.

There were some age-related statistical differences between days 14 and 21 in the Reference animals, such as for IL-2, IL-4 and IL-12, and some of these changes were also modulated by the supplementations (P < 0.05, Table 2). To date, differently to Reference animals, the adiponectin dietary intervention significantly lowered the IL-10 and IFN-γ secretion from day 21 to those present at day 14 (P < 0.05 and P < 0.01, respectively).

Cell culture supernatants from the Leptin group showed a higher concentration of IL-4, IL-2 and IL-10 (P < 0.05 *vs*. Reference group) on day 21, the first already being observed at day 14 (P < 0.05 *vs*. reference and WPC groups, Table 2). With regard to the Adiponectin group at day 14, the IL-10 secretion was increased by twice the amount of that produced by cells from animals in the Reference group (P < 0.05) and there was also a 4-fold increase in IFN-γ concentration (P < 0.01 *vs*. Reference group and P < 0.05 *vs*. WPC). At day 21, WPC supplementation showed a higher increase in IL-12p70 secretion than that observed in Reference group (P < 0.05, Table 2).

## Discussion

In previous studies, we reported for the first time the impact of leptin and adiponectin supplementations on the intestinal immune system in suckling rats^20^. Nevertheless, the influence of these adipokines on the systemic immune compartment in early life remains unexplored. To achieve this, suckling Wistar rats were supplemented with leptin, adiponectin or a WPC very rich in breast milk biological factors, during the whole suckling period. The current study demonstrates that adipokine supplementation during the suckling period is able to stimulate the systemic IS in early life. This effect is associated with an increase in the plasma Ig concentrations and changes in spleen lymphocyte composition and the pattern of cytokine secretion. However, a current limitation of this experimental design is the fact that plasmatic leptin and adiponectin levels in pups were not evaluated after supplementation. Nevertheless, previous studies described the presence of leptin in serum of supplemented-rats^21^ and adiponectin in mice^22^ confirming the absorption of both adipokines. In addition, although the most abundant form of adiponectin in human milk is the high molecular weight multimer^23^ we have supplemented the rats with the monomeric form.

Even though the supplementations were administered daily to Wistar rats from the day of birth and throughout the suckling period, they did not affect either their body weight growth. These results are in line with some previous studies where the supplementation with these adipokines did not modify body weight^20,24^. Moreover, taking into account the long-term effect of such adipokines, it has been described that leptin and adiponectin levels in breast milk during the perinatal period might be associated with a higher risk of body weight gain and overweight in the corresponding infants in later life^13,25^. This fact reinforces the important role they have in the regulation of metabolism^26^ and metabolic diseases such as obesity or diabetes^16,27^.

Although there is a study carried out by Hick *et al*.^28^ in which the mice received leptin injections for 10 days showing a decrease in thymus weight, we have not seen these effects due to the leptin supplementation during the suckling period. However, supplementations with adiponectin and WPC for 3 weeks increased the relative thymus weight. This is the first time that such an effect on the thymus by these compounds has been reported, although it has been previously described that the thymus of exclusively breastfed babies is double that of children who are fed with formula^29^. So, adiponectin, present in breast milk, might be one of the active components that increases the weight of the thymus and therefore to have a role in immunodevelopment at such level. In this regard, the similar effect induced by either adiponectin or WPC, which has high concentration of bioactive factors on the thymus, reinforces the effect of the first on this lymphoid organ. However, further studies of the possible cellular and histological changes induced in the thymus by both supplementations should be addressed.

The current plasma concentrations of inmunoglobulins in the Reference group presented in this study showed similar levels of IgG, IgA and IgM to those described previously in age-matched Lewis rats^30^. Regarding plasma IgG concentrations, neither of the three supplementations produced any effect on them. Furthermore, the four plasma rat IgG isotypes were also quantified: IgG1 and IgG2a, which have been associated with a type Th2 immune response, and IgG2b and IgG2c, related to Th1^31,32^. Suckling rats fed with leptin showed a punctual increase of IgG1 at day 14, a result that did not match with the suggested leptin effect on the switch from IgG1 to IgG2a isotype^33–35^. Moreover, animals fed with adiponectin increased IgG1 and IgG2a in the same way as WPC supplementation, suggesting an early maturation similar to that found for this positive control product with immunomaturative properties^36^. Although no one has described before the effects of adiponectin on plasma IgG isotypes, these results on adipokine increased Th2-related Ig are in line with the anti-inflammatory effects that are attributed to adiponectin^12,26,37^. In addition, it is well known that, even though there are relatively high levels of B cells at birth, the production of Ig during the postnatal period is far from that produced in adults^38^. So, the increase of IgM due to the adiponectin supplementation at both studied days and leptin at day 21 could also suggest a role of these adipokines in the early maturation of their adaptive immunity.

In the present study, we describe for the first time, to the best of our knowledge, the effect of the adipokines on the lymphocyte composition in the spleen, which is a relevant lymphoid tissue in the systemic compartment. Moreover, in all of the main spleen lymphocyte subsets in the Reference group, with the exception of B cells, there was an age-related increase in accordance with previous studies describing the development of the IS during the postnatal period^30,38,39^. In this study, none of the three supplementations altered the splenocytes’ percentage of B, Treg and TCRγδ^+^ cells. However, rats fed with leptin were able to increase the T cell proportion by increasing the T/B cells ratio at both day 14 and 21. This adipokine was also able to enhance TCRαβ^+^ and TCRαβ^+^ CD8^+^ cell proportion at both studied days. This effect strengthens the ability of this adipokine to increase these populations previously described in the intestinal compartment^20^. Moreover, the Adiponectin group increased these populations proportions at day 14. Controversially, Wilk *et al*.^40^ showed that adiponectin could negatively regulate human CD8^+^ T cells. We also found here that both adipokine supplementations increased the percentage of Th cells (TCRαβ^+^ CD4^+^) leptin at both days, and adiponectin only at day 14. This last result was in concordance with other studies that evidenced that leptin induces the proliferation of CD4^+^ T cells^41,42^.

With regard to NK and NKT cells, it has been previously described that leptin is able to increase the development and activation of NK cells both *in vitro* and *in vivo*^18,43–45^, in a similar way to what was observed in this study on rats supplemented with leptin. Although no differences were observed in the NKT population in the intestinal compartment^20^, the three supplementations increased NKT and NKT CD8^+^ cell percentages in the spleen. To better understand neonatal systemic immunity, the total proportion of cells bearing CD8^+^ was studied. Rats fed with leptin or WPC showed a remarkable increase of CD8^+^ cells proportion at the end of the suckling period. The role of leptin in enhancing CD8^+^ splenocytes was also described in mice^46^ and in the intestinal compartment of rats^20^. Moreover, human studies also demonstrate this effect *in vitro*^17,19^. In the current study, this enhancement could be attributed to the higher CD8^+^ CD62L^+^ cell percentage. The CD62L molecule is involved in lymphocyte homing^47^. The increase in the proportion of this specific population could mean that the leptin and WPC supplementations increased the arrival of lymphocytes to the spleen and, consequently, their activation. Alternatively, it could also indicate an induction of adhesion molecules in the early phase of homing to other tissues.

Another aspect studied in the spleen was the lymphocyte proliferation after *in vitro* stimulation. Although none of the splenocytes from any of the studied groups presented any change after 48 h with anti-CD3/anti-CD28 stimulation, cells from the intestinal compartment of rats supplemented with adiponectin showed an increase in lymphoproliferative capacity^20^. However, some differences were found in the cytokine secretion pattern. Overall, leptin supplementation was able to increase IL-4 at both studied days, and IL-2 and IL-10 at the end of the suckling period. Thus, the rise in IL-4 due to leptin at both ages may be linked to the enhancement in the IgG1 (Th2 isotype) rise at day 14 described before. Moreover, the IL-2 increase can be justified on the basis that there are some studies showing that leptin can enhance T CD4^+^ lymphocyte activation by stimulating the synthesis of IL-2^17,48^. Additionally, these results are in line with our previous results, which showed that the supplementation with these adipokines produced an increase of IL-2 and IL-4 by lymphocytes from mesenteric lymph nodes^20^. With regard to the IL-10 increase, leptin showed a role in the activation of B lymphocytes, inducing secretion of the anti-inflammatory and immunoregulatory cytokine IL-10 via JAK–STAT and p38MAPK–ERK1/2 signalling^49^, so the increase of the secretion of this cytokine could be due to the activation of this pathway.

Moreover, we found an increase of IL-10 and IFN-γ in those rats supplemented with adiponectin at day 14. Some human and *in vitro* studies also show the anti-inflammatory role of adiponectin, by decreasing TNF-α or enhancing IL-10 levels^15,50,51^. On the other hand, our result regarding IFN-γ did not match with those reported previously, where a decrease of this cytokine was found by adiponectin^50,52^. But it has to be mentioned that both studies were performed with other types of cells and they were conducted in human and mice.

Furthermore, an association between these adipokines and the IS has also been reported in autoimmune diseases, such as systemic lupus erythematosus or rheumatoid arthritis^34,52^. In line with this, high concentration of leptin and adiponectin has been detected in serum of patients with these diseases^16,37^. Unfortunately, the effect of these adipokines on children’s immunity or in later life remains still un-explored. In our study we showed the early maturation of the systemic IS during the suckling period due to the supplementation of leptin or adiponectin but, further studies should be addressed to elucidate whether these adipokines could have a programming effect and therefore modulate immune responses later in life.

In conclusion, supplementations with adipokines, leptin or adiponectin, during the whole suckling period involve immunomodulatory effects in the systemic immune response in rats at early life. With regard to the developing humoral response, both adipokines modulated IgG isotype proportions and also increased IgM levels, suggesting an early maturation of B cells secreting high levels of Ig, which is deficient in newborns. This study also demonstrates the impact of these adipokine supplementations on the lymphocyte composition, showing a remarkable induction of maturation of acquired immunity, particularly on T cells, by increasing the proportion of TCRαβ^+^ CD4^+^ and TCRαβ^+^ CD8^+^ subpopulations. In addition, NKT cell subset development was also promoted by both adipokines. Furthermore, supplementation of both adipokines modulated the cytokine pattern secreted by splenocytes. In summary, these changes might contribute to enhancing the immature immune system of the newborn, thereby underlining the maturative role of leptin and adiponectin in this period for enhancing early IS development.

## Materials and Methods

### Animals

Sixteen G15 pregnant Wistar rats (RjHan:WI strain) from Janvier Labs (Le Genest-Saint-Isle, France) were individually housed in cages and monitored daily. The animals were housed under controlled temperature and humidity conditions, in a 12:12 h light:dark cycle in the Faculty of Pharmacy and Food Science animal facilities in non-specific pathogen free conditions. With regard to sample size estimation, four litters were required for each group as previous studies have demonstrated a notable role of variability among litters^53^. This calculation was made by the Appraising Project Office’s programme from the Universidad Miguel Hernández de Elche (Alicante), used to provide statistically significant differences among groups, assuming that there is no dropout rate and a type I error of 0.05 (two-sided). All the experimental procedures were performed in accordance with the Guide for the Care and Use of Laboratory Animals. The study was reviewed and approved by the Ethical Committee for Animal Experimentation of the University of Barcelona (CEEA/UB ref. 220/15).

Dams were fed with commercial diet corresponding to the American Institute of Nutrition 93 M formulation (Harlan Teklad, Madison, Wisconsin, USA) and water *ad libitum*. Pregnant rats were allowed to deliver at term. The day after birth was registered as day 1 of life. Litters were assigned to the experimental groups and culled to 9 pups per lactating dam. Pups had free access to the nipples and the rat diet and they were individually identified and weighed daily. Handling was done in the same time range to avoid the influence of biological rhythms.

### Dietary supplementation

According to the oral supplementation given, suckling rats were randomly distributed into four groups (n = 4 dams with 9 pups each/group; n = 36 pups/group): the Reference, the Leptin, the Adiponectin and the Whey Protein Concentrate (WPC) groups. As WPC is rich in bioactive and immune factors it was used as a product expected to have immunomodulatory impact on neonatal rats, as suggested in previous studies^36,54^. The first 21 days of a rat’s life correspond to the lactating period of humans, thus the supplementation during the first 21 days fits the entire suckling period. Daily, pups received the supplements by oral gavage using low-capacity syringes (Hamilton Bonaduz, Bonaduz, Switzerland) adapted to oral 25- or 23-gauge gavage tubes (ASICO, Westmont, IL, USA), as previously described^36^. Litters were separated from dams half an hour before oral supplementation to allow gastric emptying. The Leptin group was supplemented with 0.7 μg/kg/day of recombinant rat leptin (PeproTech®, Rocky Hill, NJ, USA) in water; the Adiponectin group was administered with recombinant murine adiponectin (PeproTech®) solution at a dose of 35 μg/kg/day in water; the WPC group received Lacprodan® MFGM-10 (Arla Foods Ingredients Group, Diby, Denmark) at a dose of 0.3 g/kg/day in water. Administered doses of all supplementations were selected in basis of other similar studies^55–58^ and as performed in a previous approach^20^. The Reference group was administered with the same volume of water (vehicle) as the supplemented groups (10 mL/kg/day).

### Sample collection and processing

During (day 14) and at the end of the suckling period (day 21), 3 pups of each mother (12 rats/group/day) were weighed and intramuscularly anaesthetised with ketamine (9 mg/kg) (Merial Laboratories S.A., Barcelona, Spain) and xylazine (10 mg/kg) (Bayer A.G., Leverkusen, Germany). Apart from blood samples, spleen and thymus were collected and weighed under sterile conditions. Blood samples were centrifuged and plasma was kept at −20 °C for Ig determination.

### Ig quantification

Plasma IgA, IgM, IgG1, IgG2a, IgG2b and IgG2c concentrations were quantified by ProcartaPlex Rat Antibody Isotyping Panel (eBioscience, Frankfurt, Germany), following the manufacturer’s instructions. Each Ig isotype concentration was quantified according to the different detection antibodies conjugated to phycoerythrin and analysed by the Luminex MAGPIX analyser (Luminex®, Austin, TX, USA) at the Scientific and Technological Centres of the University of Barcelona (CCiT-UB). Assay sensitivity was as follows: 0.48 pg/mL for IgA; 0.02 ng/mL for IgM; 0.78 ng/mL for IgG1; 0.02 ng/mL for IgG2a; 0.11 ng/mL for IgG2b and 0.19 pg/mL for IgG2c.

### Spleen lymphocytes isolation

Splenocytes were isolated, as previously described^59^, by passing the tissue individually through a sterile 40 µm mesh cell strainer (Thermo Fisher Scientific, Barcelona, Spain). The resultant cell suspensions were submitted to the osmotic lyses of erythrocytes. Cell counting and viability were assessed by Countess^TM^ Automated Cell Counter (InvitrogenTM, Thermo Fisher Scientific). Splenocytes were then used to study their phenotype, and their proliferation capacity and cytokine production, after mitogenic stimulation.

### Immunofluorescence staining and flow cytometry analysis

For flow cytometric analysis, splenocytes (2 × 10^5^) were labelled with mouse anti-rat monoclonal antibodies (mAb) conjugated to fluorescein isothiocyanate (FITC), phycoerythrin (PE), peridininchlorophylla protein (PercP), allophycocyanin (APC) or APC-Cyanine (Cy)7, as in previous studies^60^. In this case, the antibodies used were anti-CD4, anti-CD8α, anti-CD8β, anti-TCRαβ, anti-TCRγδ, anti-NKR-P1A, anti-CD25, anti-CD45RA (BD Biosciences, San Diego, USA), anti-CD62L, anti-CD103 (Biolegend, San Diego, CA, USA), anti-TLR-4 (Novus Biologicals, Littlon, CO, USA) and anti-Foxp3 (eBioscience). After staining with standard procedures^20^, analyses were performed using a Gallios^TM^ Cytometer (Beckman Coulter Inc., Miami, FL, USA) at the CCiT-UB. All results were assessed by the Flowjo v.10 software (TreeStar, Inc., Ashland, OR, USA).

### Proliferation assay and cytokine production in stimulated splenocytes

At day 14 and 21, splenocytes (10^5^ cells/200 µL) were incubated in quadruplicate with the anti-CD3 mAb (10 µg/mL, BD Biosciences) and anti-CD28 mAb (20 µg/mL, BD Biosciences) or without stimulus in 96-well plates (TPP, Trasadingen, Switzerland). Stimulated cells (SC) and non-stimulated cells (NSC) were incubated in sterile conditions for 48 h at 37 °C and 5% CO_2_ atmosphere in a humidified chamber. At 46 h, 5-bromo- 2′-deoxyuridine was added and 2 h later plates were then centrifuged (210 *g*, 5 min) and proliferation was quantified by means of the Cell Proliferation Assay Kit (Merck Millipore, Darmstadt, Germany), following the manufacturer’s instructions. Absorbance (Abs), was measured at 450 nm on a microplate photometer (Labsystems Multiskan, Helsinki, Finland). The proliferation rate was expressed as A/B, where, A = [(Abs-SC – Abs-NSC) / Abs-NSC] for the supplemented group and the mean value of B = [(Abs-SC – Abs-NSC)/Abs-NSC] for the Reference group.

To assess cytokine production, IL-2, IL-4, IL-10, IL-12p70, IFN-γ, and TNF-α were quantified from supernatants after the above-described stimulation, also using a ProcartaPlex^®^ multiplex immunoassay (eBioscience), according to the manufacturer’s protocol and previous studies^20^. Results were analysed by the Luminex MAGPIX analyser (Luminex®) at the CCiT-UB.

### Statistical analysis

Statistics were performed by the software IBM Statistical Package for the Social Sciences (SPSS, version 22.0, Chicago, IL, USA). The Levene’s test was carried out to assess the homogeneity of variance and the Shapiro−Wilk test to evaluate the distribution of the results. Conventional one-way ANOVA test followed by the Bonferroni post hoc test was performed when there was a normal distribution and equality of variance existed. On the other hand, the results having different variance and/or different distribution were evaluated by the non-parametric Kruskal−Wallis test followed by the Mann−Whitney U post hoc test. Significant differences were established at P < 0.05.
